# Differences in clinical outcomes according to the time interval between the bridge to surgery stenting and surgery for left-sided malignant colorectal obstruction

**DOI:** 10.1186/s12957-022-02644-9

**Published:** 2022-06-03

**Authors:** Hyung-Hoon Oh, Ji-Yun Hong, Dong-Hyun Kim, Dae-Seong Myung, Sung-Bum Cho, Wan-Sik Lee, Hyun-Soo Kim, Young-Eun Joo

**Affiliations:** Department of Internal Medicine, Chonnam National University Medical School, 8 Hak-Dong, Dong-ku, Gwangju, 501-757 Republic of Korea

**Keywords:** Self-expandable metal stent, Colon cancer, Bridge to surgery interval

## Abstract

**Background:**

Self-expandable metal stent (SEMS) placement is commonly used as a bridge to surgery (BTS) for left-sided malignant colorectal obstruction (MCO). However, the optimal time interval between BTS stenting and surgery for left-sided MCO is unclear, and the results of previous studies are conflicting. This study aimed to determine the differences in clinical outcomes according to the time interval between BTS stenting and surgery in left-sided MCO.

**Methods:**

Data from 594 patients who underwent SEMS placement for MCO between January 2009 and December 2018 were reviewed. Among them, 148 patients who underwent SEMS placement as BTS treatment and curative surgery were enrolled. The enrolled patients were divided into three groups according to the interval between BTS stenting and surgery: group 1 (interval ≤2 weeks), group 2 (interval 2–3 weeks), and group 3 (interval >3 weeks).

**Results:**

Group 2 and 3 patients underwent significantly higher rates of laparoscopic surgery than those in group 1 (83.7, 81.0 vs. 53.2 %, respectively; *P*=0.003, *P*=0.003, respectively). Also, rates of stoma formation directly after resection were significantly higher in group 1 compared to groups 2 and 3 (21.3 vs 2.3, 6.9%, respectively; *P*=0.008, *P*=0.043, respectively). Bridging interval had no effect on SEMS-related complications, resection-related complications, 90-day mortality, permanent stoma formation, 3-year disease-free survival, and 3-year overall survival.

**Conclusions:**

A bridging interval of > 2 weeks between BTS stenting and surgery for left-sided MCO is preferable for lower stoma formation rates and higher rates of laparoscopic approach operation, with no difference in short-term and long-term outcomes including complication, mortality, and survival.

**Supplementary Information:**

The online version contains supplementary material available at 10.1186/s12957-022-02644-9.

## Background

Colorectal cancer is the third-most common cancer worldwide, and its incidence in patients <50 years of age has been increasing [1]. Approximately 8–29% of patients with colorectal cancer experience malignant colorectal obstruction (MCO) and require emergency decompression [2, 3]. Classically, MCO has been treated with emergency surgery with stoma formation or primary resection. However, recent studies have reported that emergency surgery is associated with higher morbidity and mortality compared to elective surgery [4, 5]. Therefore, the need for alternative treatment options has emerged.

Since Dohmoto et al. first reported a case of self-expandable metal stent (SEMS) insertion for palliation of MCO [6], numerous studies have evaluated the efficacy of SEMS insertion in MCO. SEMS insertion can be performed for one of two purposes: to act as a bridge to surgery (BTS) or for palliation. In the former case, SEMS insertion has been accepted as a viable treatment option, especially for left-sided MCO. It shows a similar overall survival (OS) rate, lower complication rates, and lower stoma formation rates compared to emergency surgery [7–9]. Decompression with SEMS insertion allows bowel preparation, medical stabilization including repair of dehydration and electrolyte abnormalities, and optimization of concomitant conditions, all of which enhance bowel condition, nutrition, and inflammatory status in patients, thereby allowing elective one-stage surgery [10, 11]. However, the optimal time interval between SEMS placement and surgery remains unclear.

In 2014, the European Society of Gastrointestinal Endoscopy (ESGE) suggested 5–10 days as an optimum time interval between SEMS placement and operation when SEMS is used as BTS; however, this was based on low-quality evidence [12]. In 2020, the updated ESGE guideline suggested an interval of approximately 2 weeks, which was also based on low-quality evidence [7]. As there is no prospective comparative study concerning the time interval between SEMS placement and surgery, the guideline was based on retrospective studies and expert opinions. After the ESGE guidelines were updated in 2020, several studies have reported the optimal timing of surgery after SEMS placement. However, the data are conflicting.

In this study, we aimed to compare the short-term and long-term clinical outcomes according to the time interval between BTS stenting and surgery in left-sided MCO.

## Materials and methods

### Patient enrollment

A total of 594 patients who underwent SEMS placement for MCO between January 2009 and December 2018 at the Chonnam National University Hwasun Hospital and Chonnam National University Hospital were retrospectively reviewed. The eligibility criteria included (1) patients who were diagnosed with colonic obstruction using a combination of clinical and radiologic signs of obstruction, (2) histologically confirmed cancer, located in the left-sided colon (defined as splenic flexure, descending colon, or sigmoid colon), and (3) patients who had SEMS placement as a bridge to curative intent surgery. Exclusion criteria were (1) stage IV cancer or non-curative resection, (2) concurrent history of other cancers, (3) neoadjuvant chemotherapy, and (4) technical or clinical failure of SEMS placement. Finally, a total of 148 patients were enrolled in this study. Enrolled patients were divided into three groups according to bridging interval: (group 1, ≤2 weeks; group 2, 2–3 weeks; and group 3, >3 weeks). The range of intervals was based on the ESGE guidelines, which recommend a bridging interval of about 2 weeks. We compared the short- and long-term outcomes of patients divided by an interval of 2 weeks.

### Procedure protocol

In our hospitals, patients with obstructive signs or symptoms such as abdominal pain, distension, and failure of gas passage underwent abdominal and chest computed tomography (CT) within 24 h of admission. Patients with signs of peritoneal irritation or hemodynamic instability were counseled to undergo surgery without stent placement. Every patient who underwent SEMS placement was assessed for technical and clinical success and complications. Technical success was defined as the successful placement of the SEMS over the obstruction site without any adverse events. Clinical success was defined as an improvement of obstructive symptoms and radiologic relief within 48 h after SEMS placement. The time for surgery was decided by the colorectal surgeons based on the patient’s general condition.

### Operation characteristics and outcome measures

Operational characteristics included type of resection, the urgency of resection (emergency vs. elective), surgical approach (open vs. laparoscopic), stoma formation directly after resection, and operation time. Short-term treatment outcomes, including SEMS-related complications, operation-related complication rate, and 90-day mortality, were analyzed. Additionally, resection-related complications were classified according to the Clavien–Dindo classification (grades I, II vs. III, IV, and V) [13]. Grade >III indicated complications requiring surgical or endoscopic intervention. Regarding long-term treatment outcomes, the primary outcome parameters were 3-year disease-free survival (DFS) and 3-year OS rates.

### Statistical analysis

Non-normally distributed variables were reported as medians (interquartile range [IQR]). The Mann–Whitney *U* test was used for comparisons between two groups and the Kruskal–Wallis test was used for comparisons between three groups. Categorical variables are expressed as frequencies and percentages. The Student’s *t* test, chi-square test, or analysis of variance was used as appropriate. Differences regarding disease-free and OS rates were assessed using the log-rank test. Using a logistic regression model, we determined predictive factors associated with recurrence and death. The Cox proportional hazards regression model with forward selection for variables that were significant in the univariate analysis for OS or DFS was used for multivariate analysis. The statistical significance was set at *P* < 0.05. All data were analyzed using Statistical Package for the Social Sciences, version 27.0 (SPSS Inc., Chicago, IL, USA).

## Results

### Baseline characteristics of enrolled patients according to bridging interval

A total of 148 patients were enrolled in this study. Forty-seven patients underwent surgery after a bridging interval of ≤2 weeks (group 1), 43 patients after 2–3 weeks (group 2), and 58 patients after >3 weeks (group 3). The baseline characteristics of the enrolled patients are summarized in Table 1. The median age was 68.0 years (IQR 55.0–78.0) in group 1, 66.0 years (IQR 56.0–76.0) in group 2, and 70.5 years (IQR 61.8–77.0) in group 3; no significant difference was found between the groups’ ages. There were also no significant differences in body mass index (BMI), American Society of Anesthesiologists (ASA) score, previous abdominal surgery history, primary tumor location, preoperative serum carcinoembryonic antigen (CEA) level, or adjuvant chemotherapy rates among the three groups. The interval from SEMS to operation was 10.0 days (IQR 8.0–12.0) in group 1, 18.0 days (IQR 17.0–20.0) in group 2, and 28.0 days (IQR 24.0–32.3) in group 3.

**Table 1:** Baseline characteristics of enrolled patients

	Total ***n*** (%)	Bridging interval			***P***-value			
		Group 1 ≤2 weeks ***n*** (%)	Group 2 2–3 weeks ***n*** (%)	Group 3 >3 weeks ***n*** (%)	Overall	≤2 weeks ***vs***. 2–3 weeks	2–3 weeks vs. >3 weeks	≤2 weeks vs. >3 weeks
Number of patients	148	47	43	58				
Male sex	86 (58.1)	28 (59.6)	31 (72.1)	27 (46.6)	**0.037**	0.269	**0.014**	0.239
Age, median years (IQR years)	68.0 (58.0–77.0)	68.0 (55.0–78.0)	66.0 (56.0–76.0)	70.5 (61.8–77.0)	0.313	0.545	0.122	0.427
BMI, median kg/m^2^ (IQR kg/m^2^)	22.2 (20.0–24.9)	22.8 (20.6–25.0)	22.0 (20.2–24.8)	22.2 (19.3–25.0)	0.846	0.634	0.844	0.620
ASA score					0.119	0.679	0.066	0.253
ASA 1–2	132 (89.2)	43 (91.5)	43 (95.3)	48 (82.8)				
ASA 3–4	16 (10.8)	4 (8.5)	2 (4.7)	10 (17.2)				
Previous abdominal surgery	43 (29.1)	12 (25.5)	13 (30.2)	18 (31.0)	0.829	0.645	1.000	0.665
Primary tumor location					0.378	0.567	0.510	0.174
Splenic flexure	9 (6.1)	4 (8.5)	1 (2.3)	4 (6.9)				
Descending colon	15 (10.1)	2 (4.3)	4 (9.3)	9 (15.5)				
Sigmoido-descending junction	11 (7.4)	2 (4.3)	4 (9.3)	5 (8.6)				
Sigmoid colon	75 (50.7)	28 (59.6)	24 (55.8)	23 (39.7)				
Recto-sigmoid junction	38 (25.7)	11 (23.4)	10 (23.3)	17 (29.3)				
Preoperative serum CEA, median ng/ml (IQR ng/ml) (*n*=146)	5.7 (3.2–13.6)	7.8 (4.3–15.3)	4.9 (2.5–9.8)	5.2 (2.7–11.7)	0.127	0.067	0.920	0.089
Adjuvant chemotherapy	116 (78.4)	38 (80.9)	35 (81.4)	43 (74.1)	0.633	1.000	0.475	0.488
Interval from SEMS to operation, median days (IQR days)	19.0 (12.0–25.8)	10.0 (8.0–12.0)	18.0 (17.0–20.0)	28.0 (24.0–32.3)	**<0.001**	**<0.001**	**<0.001**	**<0.001**
Pathologic results								
T stage					0.894	1.000	0.832	0.664
1	1 (0.7)	1 (2.1)	0 (0.0)	0 (0.0)				
3	101 (68.2)	32 (68.1)	30 (69.8)	39 (67.2)				
4	46 (31.1)	14 (29.8)	13 (30.2)	19 (32.8)				
N stage					0.139	**0.037**	0.139	0.756
0	66 (44.6)	18 (38.3)	24 (55.8)	24 (41.4)				
1	54 (36.5)	22 (46.8)	9 (20.9)	23 (39.7)				
2	28 (18.9)	7 (14.9)	10 (23.3)	11 (19.0)				
Overall stage					0.205	0.138	0.164	0.842
2	66 (44.6)	18 (38.3)	24 (55.8)	24 (41.4)				
3	82 (55.4)	29 (61.9)	19 (44.2)	34 (58.6)				
Tumor size, median cm (IQR cm)	6.5 (5.5–7.5)	6.0 (5.0–6.5)	7.0 (6.0–8.0)	7.2 (7.0–8.0)	**<0.001**	**<0.001**	0.329	**<0.001**
Histologic grade					0.225	0.105	0.648	0.501
WD/MD	143 (96.6)	47 (100.0)	40 (93.0)	56 (96.6)				
PD/Mucinous	5 (3.4)	0 (0.0)	3 (7.0)	2 (3.4)				
Lymphovascular invasion	39 (26.4)	11 (23.4)	15 (34.9)	13 (22.4)	0.316	0.253	0.184	1.000
Perinueral invasion	97 (65.5)	29 (61.7)	29 (67.4)	39 (67.2)	0.841	0.661	1.000	0.682

*IQR* Interquartile range, *BMI* Body mass index, *ASA* American Society of Anesthesiologists, *CEA* Carcinoembryonic antigen, *WD* Well-differentiated, *MD* Moderately differentiated, *PD* Poorly differentiated

### Operation characteristics

The operational characteristics are summarized in Table 2. 95.3% of group 2 and 98.3% of group 3 patients were discharged from the hospital during the bridging interval, which was significantly higher compared with group 1. Concerning urgency of resection, 10.6% of patients in group 1 underwent an emergency operation, which was significantly higher than in groups 2 and 3. Group 2 and group 3 patients underwent significantly higher rates of laparoscopic surgery than those in group 1 (83.7, 81.0 vs. 53.2%, respectively; *P*=0.003, *P*=0.003, respectively). Also, the rate of stoma formation directly after resection was significantly higher in group 1 (21.3%) compared with groups 2 (2.3%; *P*=0.008) and 3 (6.9%; *P*=0.043). There was no significant difference in the duration of surgery between the three groups.

**Table 2:** Perioperative characteristics of enrolled patients

	Total ***n*** (%)	Bridging interval			***P***-value			
		Group 1 ≤2 weeks ***n*** (%)	Group 2 2–3 weeks ***n*** (%)	Group 3 >3 weeks ***n*** (%)	Overall	≤2 weeks vs. 2–3 weeks	2–3 weeks vs. >3 weeks	≤2 weeks vs. >3 weeks
Number of patients	148	47	43	58				
Discharge from hospital during bridging interval	113 (79.0)	15 (31.9)	41 (95.3)	57 (98.3)	**<0.001**	**<0.001**	0.573	**<0.001**
Type of resection					**0.028**	0.063	0.361	**0.024**
Anterior resection	94 (63.5)	26 (55.3)	30 (69.8)	38 (65.5)				
Low anterior resection	19 (12.8)	9 (19.1)	4 (9.3)	6 (10.3)				
Left hemicolectomy	26 (17.6)	6 (12.8)	9 (21.0)	11 (19.0)				
Subtotal colectomy	4 (2.7)	4 (8.5)	0 (0.0)	0 (0.)				
Total colectomy	3 (2.0)	0 (0.0)	0 (0.0)	3 (5.2)				
Hartmann’s operation	2 (1.4)	2 (4.3)	0 (0.0)	0 (0.0)				
Urgency of resection					**0.005**	0.057	NA	**0.016**
Emergency	5 (3.4)	5 (10.6)	0 (0.0)	0 (0.0)				
Elective	143 (96.6)	42 (89.4)	43 (100.0)	58 (100.0)				
Surgical approach					**0.001**	**0.003**	0.797	**0.003**
Open	40 (27.0)	22 (46.8)	7 (16.3)	11 (19.0)				
Laparoscopic	108 (73.0)	25 (53.2)	36 (83.7)	47 (81.0)				
Stoma directly after resection	16 (10.8)	10 (21.3)	1 (2.3)	4 (6.9)	**0.010**	**0.008**	0.391	**0.043**
Operation time, median min (IQR min)	150.0 (120.0–198.8)	160.0 (125.0–205.0)	149.0 (110.0–190.0)	150.0 (125.0–201.3)	0.487	0.279	0.308	0.976

*IQR* Interquartile range

### Short-term treatment outcomes

The short-term treatment outcomes are summarized in Table 3. 14.9% of group 1 patients had SEMS-related complications, including perforation (4.3%) and migration (10.6%). Seven percent of group 2 patients had SEMS-related complications, and 6.9% of group 3 patients had SEMS-related complications. Rates of operation-related complications within 90 days were 38.3% in group 1, 41.9% in group 2, and 37.9% in group 3, which were not significantly different. Postoperative complications classified according to the Clavien-Dindo classification [13] showed no significant difference among the three groups. There was no difference in a post-resection hospital stay or 90-day mortality between the groups. In addition, the interval from resection to adjuvant chemotherapy was not significantly different among the groups.

**Table 3:** Short-term treatment outcomes according to bridging interval

	Bridging interval				***P***-value			
	Total ***n*** (%)	Group 1 ≤2 weeks ***n*** (%)	Group 2 2–3 week ***n*** (%)	Group 3 >3 weeks ***n*** (%)	Overall	≤2 weeks vs. 2–3 weeks	2–3 weeks vs. >3 weeks	≤2 weeks vs. >3 weeks
Number of patients	148	47	43	58				
SEMS-related complication	14 (9.5)	7 (14.9)	3 (7.0)	4 (6.9)	0.305	0.320	0.859	0.408
Perforation	2 (1.4)	2 (4.3)	0 (0.0)	0 (0.0)	0.182	0.495	NA	0.198
Migration	8 (5.4)	5 (10.6)	1 (2.3)	2 (3.4)	0.214	0.206	1.000	0.238
Pain	1 (0.7)	0 (0.0)	1 (2.3)	0 (0.0)	0.291	0.478	0.426	NA
Bleeding	1 (0.7)	0 (0.0)	1 (2.3)	0 (0.0)	0.291	0.478	0.426	NA
Fever	2 (1.4)	0 (0.0)	0 (0.0)	2 (3.4)	0.207	NA	0.334	0.326
Operation related complications within 90 days	58 (39.2)	18 (38.3)	18 (41.9)	22 (37.9)	0.932	0.830	0.837	1.000
Clavien–Dindo classification (*n*=58)					0.904	1.000	1.000	0.673
Grade 1–2	49 (84.5)	16 (88.9)	15 (83.3)	18 (81.8)				
Grade 3–5	9 (15.5)	2 (11.1)	3 (16.7)	4 (18.2)				
Post-resection hospital stays, median days (IQR days)	8.0 (7.0–11.8)	9.0 (7.0–13.0)	8.0 (7.0–11.0)	8.0 (7.0–10.0)	0.159	0.210	0.652	0.059
90-day mortality	2 (1.4)	0 (0.0)	0 (0.0)	2 (3.4)	0.334	NA	0.506	0.501
Interval from resection to adjuvant chemotherapy, median days (IQR days) (*n*=116)	34.5 (30.0–41.0)	32.0 (29.0–41.3)	36.0 (31.0–41.0)	34.0 (30.0–39.0)	0.470	0.331	0.269	0.666

*SEMS* Self-expandable metal stent, *IQR* Interquartile range

### Long-term treatment outcomes

The long-term treatment outcomes are summarized in Table 4. The median follow-up duration was 45.0 months (IQR 27.0–60.0) in group 1, 45.0 months (IQR 31.0–59.0 in group 2, and 31.5 months (15.5–59.0) in group 3. No significant difference was observed in permanent stoma formation. The median DFS was 45.0 months (IQR 11.0–60.0) in group 1, 39.0 months (IQR 12.–58.0) in group 2, and 31.5 months (IQR 13.0–57.5) in group 3. Three-year DFS rates for groups 1, 2, and 3 were 75.3%, 65.7%, and 78.2%, respectively. Median OS was 43.0 months (IQR 23.0–70.0) in group 1, 42.0 months (IQR 25.0–62.0) in group 2, and 40.5 months (25.5–75.3) in group 3. Three-year OS rates for groups 1, 2, and 3 were 82.1%, 77.3%, and 79.0%, respectively. There was no significant difference in DFS, 3-year DFS, OS, or 3-year OS between the three groups.

**Table 4:** Long-term treatment outcomes according to bridging interval

	Bridging interval				***P***-value			
	Total ***n*** (%)	Group 1 ≤2 weeks ***n*** (%)	Group 2 2–3 weeks ***n*** (%)	Group 3 >3 weeks ***n*** (%)	Overall	≤2 weeks vs***.*** 2–3 weeks	2–3 weeks vs. >3 weeks	≤2 weeks vs. >3 weeks
Number of patients	148	47	43	58				
Follow-up, median months (IQR months)	41.0 (23.0–59.0)	45.0 (27.0–60.0)	45.0 (31.0–59.0)	31.5 (15.5–59.0)	0.135	0.960	0.082	0.100
Duration of stoma, median days (IQR days) (*n*=16)	187.5 (114.5–260.0)	191.0 (142.3–267.5)	20.0 (20.0–20.0)	210.0 (18.5–487.0)	0.295	0.182	0.667	0.768
Permanent stoma at time of last follow–up	5 (3.4)	2 (4.3)	0 (0.0)	3 (5.2)	0.385	0.495	0.259	1.000
Recurrence	38 (25.7)	12 (25.5)	15 (34.9)	11 (19.0)	0.192	0.365	0.106	0.481
Death	47 (31.8)	17 (36.2)	12 (27.9)	18 (31.0)	0.716	0.500	0.827	0.678
Disease-free survival, median months (IQR days)	38.0 (13.0–59.0)	45.0 (11.0–60.0)	39.0 (12.0–58.0)	31.5 (13.0–57.5)	0.398	0.488	0.611	0.167
3-year disease free survival (%)	73.5	75.3	65.7	78.2	0.281	0.321	0.121	0.608
Overall survival, median months (IQR days)	42.0 (25.0–71.5)	43.0 (23.0–70.0)	42.0 (25.0–62.0)	40.5 (25.5–75.3)	0.962	0.811	0.966	0.816
3-year overall survival (%)	79.4	82.1	77.3	79.0	0.718	0.474	0.667	0.623

*IQR* Interquartile range

### Univariate and multivariate logistic regression analyses of predictive factors for recurrence and death

Univariate and multivariate logistic regression analyses revealed no significant difference between recurrence and death in terms of the interval from SEMS insertion to resection. The presence of metastatic lymph nodes was associated with recurrence, with a hazard ratio of 1.387 (1.155–1666, *P*<0.001). In addition, age, stoma formation directly after resection, adjuvant chemotherapy, overall pathologic stage, metastatic lymph nodes, and perineural invasion were significantly associated with death (Table 5).

**Table 5:** Univariate and multivariate logistic regression analyses of predictive factors for recurrence and death

	Recurrence				Death			
	Uni-variate analysis		Multi-variate analysis		Uni-variate analysis		Multi-variate analysis	
	HR (95% CI)	***P***-value	HR (95% CI)	***P***-value	HR (95% CI)	***P***-value	HR (95% CI)	***P***-value
Male sex	0.855 (0.406–1.799)	0.680			0.846 (0.421–1.702)	0.639		
Age, years	0.980 (0.952–1.009)	0.175			1.037 (1.005–1.069)	**0.022**	1.047 (1.000–1.096)	**0.044**
ASA 3–4	0.381 (0.082–1.760)	0.216	0.186 (0.022–1.586)	0.124	0.690 (0.210–2.265)	0.541		
Preoperative serum CEA, ng/ml	1.029 (1.004–1.054)	**0.020**	1.024 (0.995–1.053)	0.101	1.035 (1.009–1.061)	**0.009**	1.027 (0.991–1.063)	0.142
Interval from SEMS to resection, days	0.977 (0.939–1.017)	0.252			1.012 (0.978–1.047)	0.492		
Stoma directly after resection	2.879 (0.967–8.573)	0.057			5.189 (1.662–16.202)	**0.005**	6.617 (1.287–34.009)	**0.024**
Adjuvant chemotherapy	0.579 (0.248–1.351)	0.579	0.404 (0.149–1.094)	0.075	0.218 (0.096–0.496)	**<0.001**	0.151 (0.044–0.515)	**0.003**
Overall pathologic stage III	4.183 (1.761–9.935)	**0.001**			2.888 (1.365–6.107)	**0.006**	5.243 (1.548–17.757)	**0.008**
PD/Mucinous cancer	4.629 (0.743–28.835)	0.101			9.302 (1.010–85.672)	**0.049**	9.835 (0.770–125.638)	0.079
Metastatic LN, n	1.385 (1.172–1.636)	**<0.001**	1.387 (1.155–1.666)	**<0.001**	1.172 (1.046–1.313)	**0.006**	1.185 (1.004–1.400)	**0.045**
Lymphovascular invasion	2.337 (1.058–5.161)	**0.036**			1.054 (0.699–3.234)	0.296		
Perineural invasion	3.692 (1.426–9.559)	**0.007**			1.027 (0.495–2.131)	0.942	0.271 (0.093–0.790)	**0.017**

*ASA* American Society of Anesthesiologists, *CEA* Carcinoembryonic antigen, *SEMS* Self-expandable metal stent, *PD* Poorly differentiated, *LN* Lymph node, *HR* Hazard ratio

## Discussion

Patients with MCO often require emergency surgery. It often requires multiple-stage surgery as well as the formation of a stoma, which can reduce the patient’s quality of life severely [14]. Endoscopic decompression with SEMS can turn emergency surgery into a one-stage elective procedure, making it a viable option [7]. A number of studies have increased our understanding of SEMS as a BTS throughout the last decade. However, information on the appropriate time interval between stenting and surgery, as well as its influence, is lacking. There have been several studies recommending the time interval between SEMS placement and operation, but the results are conflicting and have limitations in several studies.

First, some studies recommended delayed surgery after SEMS placement. Akihisa et al. reported that an interval of <15 days was associated with postoperative complications after analyzing 47 patients [15]. Lee et al. reported that the rates of anastomotic leakage were significantly higher in patients who had <10 days interval between SEMS insertion and surgery [16]. Marnix et al. analyzed 168 patients with left-sided MCO, which included 94 patients who underwent SEMS insertion as a BTS. Patients were divided into three groups (ER group: emergency resection; early group; early BTS with <4 weeks interval; late group; late BTS with >4 weeks interval). A 90-day mortality was higher in the early group compared with the late group, and the late group had higher a 5-year recurrence-free survival rate than the late group. Surgical resection after a 4-week interval was an independent prognostic factor for OS. Therefore, a BTS interval of >4 weeks after SEMS placement was suggested for better short-term and long-term outcomes [17].

Other studies have suggested early surgery following the SEMS placement. A retrospective multicenter study performed by Malene et al. reported that time to resection > 18 days was associated with an increased risk of recurrence [18]. Kye et al. analyzed 174 patients who underwent surgery after SEMS placement for MCO. Patients were divided into three groups based on the interval between SEMS placement and surgery (group 1, ≤7 days; group 2, 8–14 days; and group 3, >14 days). The recurrence rate was significantly lower in group 1 compared with groups 2 and 3. The time interval between SEMS placement and surgery was an independent risk factor for disease-free survival. The authors therefore suggested an interval within 7 days to improve long-term outcome [19]. However, in the present study, the interval from SEMS to resection was not associated with recurrence nor death according to univariate and multivariate logistic regression analysis. Lim et al. reported that patients who underwent surgery within 2 weeks interval from SEMS placement had lower systemic recurrence rates after analyzing 53 patients [20]. Lastly, Sato et al. reported an interval longer than 16 days was an independent risk factor for poor relapse-free survival. An interval of >35 days was an independent risk factor for postoperative complications [21].

In the present study, short-term treatment outcomes, including operation-related complications within 90 days and 90-day mortality, were not statistically significantly different between the three groups. In addition, the interval from resection to adjuvant chemotherapy was not significantly different among the three groups. Although the difference was not statistically significant, group 1 had higher rates of SEMS-related complications, including perforation. In group 3, two patients had migration of the stent 10 days after stent insertion. Additionally, fever was documented 3 days after stent insertion in two patients who were cured with the use of antibiotics. In conclusion, all complications occurred within 3 weeks of SEMS insertion in the present study.

Long-term treatment outcomes, including recurrence, death, 3-year disease-free survival rate, and OS rate, were not significantly different between the three groups. This may be due to a small number of patients in each group. We also analyzed the short-term and long-term treatment outcomes of patients subdivided into two groups with an interval of 14 days (group 1: bridging interval <14 days, group 2: bridging interval ≥14 days), as some studies recommended delayed surgery because of high rates of operation-related complications when surgery was performed 10 to 15 days after SEMS insertion [15, 16]. However, there was no significant difference in short-term outcomes, including SEMS-related complication rates and resection-related complications within 90 days between the two groups. Moreover, there was no significant difference in the 3-year disease-free survival and OS rates between the two groups (Additional file 1).

Concerning the surgical approach (open vs. laparoscopy), a bridging interval of **≤**2 weeks was associated with higher open surgery rates than bridging intervals of >2 weeks. Even after the exclusion of emergently operated patients due to stent-related complications, the result was similar. Previously, an interval of <16 days or <4 weeks has been associated with higher open surgery rates [17, 21]. Other studies reported no significant difference in the surgical approach according to bridging intervals. However, those studies showed a tendency for higher rates of open surgery in early surgery [20, 22].

There are only a few studies on stoma formation rates according to bridging intervals. Patients are very reluctant to undergo stoma formation as it compromises normal activity and can even result in psychological problems [23]. In a previous study, a bridging interval of 5–10 days was associated with a higher stoma formation rate compared with a bridging interval >10 days [22]. Others reported that the bridging interval was not significantly associated with the stoma formation rate [20]. In the present study, the stoma formation rate was significantly higher in the patients who had a bridging interval of **≤**2 weeks. Even after removal of emergency-operated patients, group 1 still had higher stoma formation rates compared to group 2 (14.3 vs 2.3%, *p* value= 0.045).

The limitation of this study is that it is prone to selection bias because of its retrospective design. Therefore, this study should be interpreted with caution, and larger prospective studies are required to validate our findings.

## Conclusion

A bridging interval of > 2 weeks between BTS stenting and surgery for left-sided MCO was significantly lower stoma formation rates and higher rates of laparoscopic approach operation than those of a bridging interval of <2 weeks. However, the bridging interval between BTS stenting and surgery was not associated with short-term and long-term outcomes including complication, mortality, and survival. Therefore, a bridging interval of > 2 weeks between BTS stenting and surgery for left-sided MCO is preferable for lower stoma formation rates and higher rates of laparoscopic approach operation.

## Supplementary Information

**Additional file 1.** Comparison of treatment outcomes between bridging interval of <14 days and ≥14 days.

## Data Availability

All data generated or analyzed during this study are included in this published article.

## Abbreviations

BTS: Bridge to surgery
CT: Computed tomography
IQR: Interquartile range
MCO: Malignant colorectal obstruction
OR: Odds ratio
OS: Overall survival
SEMS: Self-expandable metal stents

## References

[1] Siegel RL, Miller KD, Jemal A (2020). Cancer statistics, 2020. CA Cancer J Clin.

[2] Ohman U (1982). Prognosis in patients with obstructing colorectal carcinoma. Am J Surg.

[3] Carraro PG, Segala M, Cesana BM, Tiberio G (2001). Obstructing colonic cancer: failure and survival patterns over a ten-year follow-up after one-stage curative surgery. Dis Colon Rectum..

[4] Tekkis PP, Kinsman R, Thompson MR, Stamatakis JD (2004). The Association of Coloproctology of Great Britain and Ireland study of large bowel obstruction caused by colorectal cancer. Ann Surg.

[5] Hsu TC (2005). Comparison of one-stage resection and anastomosis of acute complete obstruction of left and right colon. Am J Surg.

[6] Dohmoto M. New method: endoscopic implantation of rectal stent in palliative treatment of malignant stenosis. Endosc Dig. 1991;3:1507–12.

[7] van Hooft JE, Veld JV, Arnold D, Beets-Tan RGH, Everett S, Götz M (2020). Self-expandable metal stents for obstructing colonic and extracolonic cancer: European Society of Gastrointestinal Endoscopy (ESGE) Guideline - Update 2020. Endoscopy..

[8] Amelung FJ, Burghgraef TA, Tanis PJ, van Hooft JE, Ter Borg F, Siersema PD (2018). Critical appraisal of oncological safety of stent as bridge to surgery in left-sided obstructing colon cancer; a systematic review and meta-analysis. Crit Rev Oncol Hematol.

[9] Amelung FJ, Borstlap WAA, Consten ECJ, Veld JV, van Halsema EE, Bemelman WA (2019). Propensity score-matched analysis of oncological outcome between stent as bridge to surgery and emergency resection in patients with malignant left-sided colonic obstruction. Bri J Surg.

[10] Arezzo A, Passera R, Lo Secco G, Verra M, Bonino MA, Targarona E (2017). Stent as bridge to surgery for left-sided malignant colonic obstruction reduces adverse events and stoma rate compared with emergency surgery: results of a systematic review and meta-analysis of randomized controlled trials. Gastrointest Endosc..

[11] Allievi N, Ceresoli M, Fugazzola P, Montori G, Coccolini F, Ansaloni L (2017). Endoscopic stenting as bridge to surgery versus emergency resection for left-sided malignant colorectal obstruction: an updated meta-analysis. Int J Surg Oncol..

[12] van Hooft JE, van Halsema EE, Vanbiervliet G, Beets-Tan RG, DeWitt JM, Donnellan F (2014). Self-expandable metal stents for obstructing colonic and extracolonic cancer: European Society of Gastrointestinal Endoscopy (ESGE) Clinical Guideline. Endoscopy..

[13] Dindo D, Demartines N, Clavien PA (2004). Classification of surgical complications: a new proposal with evaluation in a cohort of 6336 patients and results of a survey. Ann Surg.

[14] Frago R, Ramirez E, Millan M, Kreisler E, del Valle E, Biondo S (2014). Current management of acute malignant large bowel obstruction: a systematic review. Am J Surg.

[15] Matsuda A, Miyashita M, Matsumoto S, Sakurazawa N, Kawano Y, Yamada T (2018). Optimal interval from placement of a self-expandable metallic stent to surgery in patients with malignant large bowel obstruction: a preliminary study. Surg Laparosc Endosc Percutan Tech..

[16] Lee GJ, Kim HJ, Baek JH, Lee WS, Kwon KA (2013). Comparison of short-term outcomes after elective surgery following endoscopic stent insertion and emergency surgery for obstructive colorectal cancer. Int J Surg (London, England)..

[17] de Roos MAJ, Hugen N, Hazebroek EJ, Spillenaar Bilgen EJ (2021). Delayed surgical resection of primary left-sided obstructing colon cancer is associated with improved short- and long-term outcomes. J Surg Oncol..

[18] Broholm M, Kobborg M, Frostberg E, Jeppesen M, Gögenür I (2017). Delay of surgery after stent placement for resectable malignant colorectal obstruction is associated with higher risk of recurrence. Int J Colorectal Dis..

[19] Kye BH, Kim JH, Kim HJ, Lee YS, Lee IK, Kang WK (2020). The optimal time interval between the placement of self-expandable metallic stent and elective surgery in patients with obstructive colon cancer. Sci Rep..

[20] Lim T, Tham HY, Yaow CYL, Tan IJ, Chan DKH, Farouk R (2021). Early surgery after bridge-to-surgery stenting for malignant bowel obstruction is associated with better oncological outcomes. Surg Endosc..

[21] Sato R, Oikawa M, Kakita T, Okada T, Abe T, Yazawa T, et al. A longer interval after stenting compromises the short- and long-term outcomes after curative surgery for obstructive colorectal cancer. Surg Today. 2021;52(4):681–89. 10.1007/s00595-021-02385-4 34648067

[22] Veld JV, Kumcu A, Amelung FJ, Borstlap WAA, Consten ECJ, Dekker JWT (2021). Time interval between self-expandable metal stent placement or creation of a decompressing stoma and elective resection of left-sided obstructive colon cancer. Endoscopy..

[23] Ayaz-Alkaya S (2019). Overview of psychosocial problems in individuals with stoma: a review of literature. Int Wound J..

