# Supplementary material for: Differences in clinical outcomes according to the time interval between the bridge to surgery stenting and surgery for left-sided malignant colorectal obstruction
Source: World J Surg Oncol. 2022 Jun 3;20:178. doi: 10.1186/s12957-022-02644-9 (PMC9164395; doi:10.1186/s12957-022-02644-9)
Supplement: Supplementary file 1 — Additional file 1. Comparison of treatment outcomes between bridging interval of <14 days and ≥14 days. [file 12957_2022_2644_MOESM1_ESM.docx]

**Additional file 1. Comparison of treatment outcomes between bridging interval of <14 days and ≥14 days**

|  |  | **Bridging interval, days** | |  |
| --- | --- | --- | --- | --- |
|  | **Total (n=148)** | **<14 (n=40)** | **≥14 (n=108)** | **P-value** |
| **Baseline characteristics** |  |  |  |  |
| Male sex | 86 (58.1) | 24 (60.0) | 62 (57.4) | 0.852 |
| Age, median years (IQR years) | 68.0 (58.0–77.0) | 66.0 (55.0–78.0) | 69.0 (59.5–77.0) | 0.420 |
| BMI, median kg/m^2^ (IQR kg/m^2^) | 22.2 (20.0–24.9) | 22.8 (20.8–25.0) | 22.0 (19.7–24.9) | 0.451 |
| ASA score |  |  |  | 1.000 |
| ASA 1–2 | 132 (89.2) | 36 (90.0) | 96 (88.9) |  |
| ASA 3–4 | 16 (10.8) | 4 (10.0) | 12 (11.1) |  |
| Comorbidity | 96 (64.9) | 26 (65.0) | 70 (64.8) | 1.000 |
| Previous abdominal surgery | 43 (29.1) | 9 (22.5) | 34 (31.5) | 0.316 |
| Primary tumor location |  |  |  | 0.096 |
| Splenic flexure | 9 (6.1) | 4 (10.0) | 5 (4.6) |  |
| Descending colon | 15 (10.1) | 2 (5.0) | 13 (12.0) |  |
| Sigmoido-descending junction | 11 (7.4) | 2 (5.0) | 9 (8.3) |  |
| Sigmoid colon | 75 (50.7) | 26 (65.0) | 49 (45.4) |  |
| Recto-sigmoid junction | 38 (25.7) | 6 (15.0) | 32 (29.60 |  |
| Preoperative serum CEA, median ng/ml (IQR ng/ml) (n=146) | 5.7 (3.2–13.6) | 7.8 (4.4–15.6) | 5.1 (2.7–10.8) | **0.036** |
| Interval from SEMS to resection, median days (IQR days) | 19.0 (12.0–25.8) | 10.0 (7.0–11.8) | 22.0 (18.0–28.8) | **<0.001** |
|  |  |  |  |  |
| **Procedural characteristics** |  |  |  |  |
| Discharge from hospital during bridging interval | 113 (76.4) | 8 (20.0) | 105 (97.2) | **<0.001** |
| Urgency of resection |  |  |  | **0.001** |
| Emergency | 5 (3.4) | 5 (12.5) | 0 (0.0) |  |
| Elective | 143 (96.6) | 35 (87.5) | 108 (100.0) |  |
| Surgical approach |  |  |  | **<0.001** |
| Open | 40 (27.0) | 22 (55.0) | 18 (16.7) |  |
| Laparoscopic | 108 (73.0) | 18 (45.0) | 90 (83.3) |  |
| Conversion (n=108) | 11 (10.2) | 4 (22.2) | 7 (7.8) | 0.084 |
| Primary anastomosis | 146 (98.6) | 38 (95.0) | 108 (100.0) | 0.072 |
| Stoma directly after resection | 16 (10.8) | 8 (20.0) | 7 (6.5) | **0.028** |
| Operation time, median min (IQR min) | 150.0 (120.0–198.8) | 147.5 (120.0–197.5) | 152.5 (121.3–198.8) | 0.641 |
|  |  |  |  |  |
| **Short-term treatment outcomes** |  |  |  |  |
| SEMS related complication | 16 (10.8) | 6 (15.0) | 10 (9.3) | 0.373 |
| Perforation | 2 (1.4) | 2 (5.0) | 0 (0.0) | 0.072 |
| Migration | 8 (5.4) | 4 (10.0) | 4 (3.7) | 0.212 |
| Pain | 1 (0.7) | 0 (0.0) | 1 (0.9) | 1.000 |
| Bleeding | 1 (0.7) | 0 (0.0) | 1 (0.9) | 1.000 |
| Fever | 4 (2.7) | 0 (0.0) | 4 (3.7) | 0.574 |
| Resection related complications within 90 days | 58 (39.2) | 17 (42.5) | 41 (38.0) | 0.705 |
| Anastomotic leakage | 2 (3.4) | 0 (0.0) | 2 (4.9) | 1.000 |
| Wound infection | 15 (25.9) | 7 (41.2) | 8 (19.5) | 0.107 |
| Intra-abdominal infection | 9 (15.5) | 0 (0.0) | 9 (22.0) | **0.047** |
| Fascial dehiscence | 3 (5.2) | 0 (0.0) | 3 (7.3) | 0.548 |
| Urinary retention | 9 (15.5) | 1 (5.9) | 8 (19.5) | 0.258 |
| Ileus | 7 (12.1) | 1 (5.9) | 6 (14.6) | 0.661 |
| Pneumonia | 6 (10.3) | 2 (11.8) | 4 (9.8) | 1.000 |
| Colitis | 3 (5.2) | 3 (17.6) | 0 (0.0) | **0.022** |
| Fever | 8 (13.8) | 2 (11.8) | 6 (14.6) | 1.000 |
| Others | 3 (5.2) | 1 (5.9) | 2 (4.9) | 1.000 |
| Clavien–Dindo classification (n=58) |  |  |  | 1.000 |
| Grade 1–2 | 49 (84.5) | 15 (88.2) | 34 (82.9) |  |
| Grade 3–5 | 9 (15.5) | 2 (11.8) | 7 (17.1) |  |
| Post-resection hospital stay, median days (IQR days) | 8.0 (7.0–11.8) | 9.0 (7.0–13.8) | 8.0 (7.0–10.0) | 0.063 |
| 90-day mortality | 2 (1.4) | 0 (0.0) | 2 (1.9) | 1.000 |
| Adjuvant chemotherapy | 116 (78.4) | 34 (85.0) | 82 (75.9) | 0.269 |
| Interval from resection to adjuvant chemotherapy, median days (IQR days) (n=116) | 34.5 (30.0–41.0) | 32.0 (28.5–43.0) | 35.0 (30.0–40.0) | 0.497 |
|  |  |  |  |  |
| **Long-term treatment outcomes** |  |  |  |  |
| Follow-up, median months (IQR months) | 41.0 (23.0–59.0) | 46.0 (31.5–59.8) | 38.5 (17.0–59.0) | 0.158 |
| Duration of stoma, median days (IQR days) (n=16) | 187.5 (114.5–260.0) | 217.0 (137.0–265.0) | 177.0 (51.0–247.5) | 0.527 |
| Permanent stoma at time of last follow-up | 5 (3.4) | 1 (2.5) | 4 (3.7) | 1.000 |
| Recurrence | 38 (25.7) | 9 (22.5) | 29 (26.9) | 0.675 |
| Death | 47 (31.8) | 13 (32.5) | 34 (31.5) | 1.000 |
| Disease free survival, median months (IQR months) | 38.0 (13.0–59.0) | 48.0 (28.0–59.8) | 34.0 (11.3–58.0) | 0.053 |
| Overall survival, median months (IQR months) | 42.0 (25.0–71.5) | 42.5 (25.0–69.3) | 41.5 (25.0–72.8) | 0.888 |
| 3-year Disease free survival (%) | 73.5 | 79.3 | 71.3 | 0.381 |
| 3-year Overall survival (%) | 79.4 | 86.4 | 76.9 | 0.892 |

IQR, interquartile range; ASA, American Society of Anesthesiologists; CEA, carcinoembryonic antigen; SEMS, self-expandable metal stent
